# Supplementary material for: Efficacy and Safety of the Genistein Nutraceutical Product Containing Vitamin E, Vitamin B3, and Ceramide on Skin Health in Postmenopausal Women: A Randomized, Double-Blind, Placebo-Controlled Clinical Trial
Source: J Clin Med. 2023 Feb 7;12(4):1326. doi: 10.3390/jcm12041326 (PMC9963595; doi:10.3390/jcm12041326)
Supplement: Supplementary file 1 [file jcm-12-01326-s001.zip › jcm-2188971-supplementary.pdf]

Table S1. Participants' satisfaction with the product

Parameters	Intention-to-treat Analysis			Per-protocol Analysis		
	**GEN Group (n = 25)**	**PLA Group (n = 25)**	***p*-Value**	**GEN Group (n = 23)**	**PLA Group (n = 22)**	***p*-Value**
Serum formulation	9.72 ± 0.54	9.16 ± 1.03	0.021	9.70 ± 0.56	9.18 ± 1.10	0.058
Wrinkle reduction	9.36 ± 0.64	8.56 ± 1.64	0.030	9.39 ± 0.66	8.50 ± 1.74	0.033
Hydration	9.60 ± 0.58	9.24 ± 1.20	0.183	9.57 ± 0.59	9.23 ± 1.27	0.265
Overall efficacy	9.64 ± 0.57	8.88 ± 1.33	0.013	9.61 ± 0.58	8.86 ± 1.42	0.031
Safety profile	9.64 ± 0.57	9.52 ± 0.77	0.534	9.61 ± 0.58	9.55 ± 0.80	0.763
Whether to recommend the product to friends and family	9.68 ± 0.63	9.20 ± 1.29	0.103	9.65 ± 0.65	9.18 ± 1.37	0.154
